# N-Acetyl Cysteine Overdose Inducing Hepatic Steatosis and Systemic Inflammation in Both Propacetamol-Induced Hepatotoxic and Normal Mice

**DOI:** 10.3390/antiox10030442

**Published:** 2021-03-12

**Authors:** Gunn-Guang Liou, Cheng-Chi Hsieh, Yi-Ju Lee, Pin-Hung Li, Ming-Shiun Tsai, Chi-Ting Li, Sue-Hong Wang

**Affiliations:** 1 Institute of Molecular Biology, Academia Sinica, No. 128, Academia Road, Section 2, Nankang, Taipei 11529, Taiwan; bogun@gate.sinica.edu.tw; 2 Office of Research and Development, College of Medicine, National Taiwan University, No. 1, Renai Road, Sec. 1, Zhongzheng, Taipei 10051, Taiwan; 3 Department of Biomedical Sciences, Chung Shan Medical University, No. 110, Sec.1, Jianguo N. Road, Taichung 40201, Taiwan; f0923115139@gmail.com (C.-C.H.); a0956566933@gmail.com (P.-H.L.); a666699999212@gmail.com (C.-T.L.); 4 Institute of Medicine, Chung Shan Medical University, No. 110, Sec.1, Jianguo N. Road, Taichung 40201, Taiwan; yijulee@csmu.edu.tw; 5 Department of Food Science and Biotechnology, Da-Yeh University, No. 168, University Rd., Dacun, Changhua 51591, Taiwan; tsaims1@mail.dyu.edu.tw; 6 Department of Medical Research, Chung Shan Medical University Hospital, No. 110, Sec.1, Jianguo N. Road, Taichung 40201, Taiwan

**Keywords:** acetaminophen overdose, N-acetyl cysteine overdose, two inbred mouse strains, hepatic inflammation, systemic inflammation, microvesicular steatosis

## Abstract

Acetaminophen (APAP) overdose induces acute liver damage and even death. The standard therapeutic dose of N-acetyl cysteine (NAC) cannot be applied to every patient, especially those with high-dose APAP poisoning. There is insufficient evidence to prove that increasing NAC dose can treat patients who failed in standard treatment. This study explores the toxicity of NAC overdose in both APAP poisoning and normal mice. Two inbred mouse strains with different sensitivities to propacetamol-induced hepatotoxicity (PIH) were treated with different NAC doses. NAC therapy decreased PIH by reducing lipid oxidation, protein nitration and inflammation, and increasing glutathione (GSH) levels and antioxidative enzyme activities. However, the therapeutic effects of NAC on PIH were dose-dependent from 125 (N125) to 275 mg/kg (N275). Elevated doses of NAC (400 and 800 mg/kg, N400 and N800) caused additional deaths in both propacetamol-treated and normal mice. N800 treatments significantly decreased hepatic GSH levels and induced inflammatory cytokines and hepatic microvesicular steatosis in both propacetamol-treated and normal mice. Furthermore, both N275 and N400 treatments decreased serum triglyceride (TG) and induced hepatic TG, whereas N800 treatment significantly increased interleukin-6, hepatic TG, and total cholesterol levels. In conclusion, NAC overdose induces hepatic and systemic inflammations and interferes with fatty acid metabolism.

## 1. Introduction

Acetaminophen (APAP) is one of the most commonly used antipyretic and analgesic drugs. APAP is safe and effective at normal therapeutic doses [1], but if used in excess can cause severe necrosis of liver cells [2] and even kidney damage [3]. Severe overdose of APAP can lead to death in humans and laboratory animals [2,4]. About 5–10% of APAP is metabolized by cytochrome P450 2E1 to produce N-acetyl-p-benzoquinone imine (NAPQI). NAPQI normally combines with glutathione (GSH) in the liver to be excreted in the urine. However, if there is an excessive intake of APAP, limited GSH is consumed, and the remaining NAPQI combines with proteins in the cytoplasm and mitochondria to cause lipid peroxidation. The covalent modifications of mitochondrial proteins cause mitochondrial permeability transition followed by mitochondrial dysfunction. The dramatic increase in oxidative stress, DNA fragmentation, and death of hepatocytes induce liver damage or failure [5].

At present, only N-acetyl cysteine (NAC) is a standard therapeutic agent for APAP-induced hepatotoxicity. The main mechanism of NAC detoxification is the provision of NAC as the raw material for GSH synthesis. Furthermore, NAC is also a reactant of mitochondrial energy metabolism [6]. Although NAC has high efficacy in reducing mortality due to APAP poisoning, some APAP overdose patients treated with standard NAC dose within 8 h experience liver damage, indicating that the dose is insufficient or ineffective. A previous report has demonstrated that increasing NAC dose reduces hepatotoxicity in patients suffering from high-level APAP toxicity [7]. However, there is no sufficient evidence to show that increasing NAC dose improves its therapeutic effect [8]. Furthermore, NAC dosing errors lead to serious side effects and even death in patients with APAP overdose [9]. In phase I pharmacokinetic and pharmacodynamic studies of NAC, major toxicities have included unpleasant taste and gastrointestinal disturbances. However, this study was early terminated due to excessive toxicity [10]. However, the toxicological mechanism of NAC overdose is not clear in both APAP overdose patients or healthy persons using single or repeated NAC doses. Therefore, we use mouse models to study the toxicological mechanisms of NAC overdose in both APAP overdose and normal mice.

Mice are most commonly used to study APAP metabolism and toxicity, as their APAP metabolic mechanism is most similar to that of humans [11]. However, in literature studies, the lethal dose and time of APAP and the therapeutic dose of NAC are very inconsistent, partly due to differences in used mouse strains. This has resulted in different effective doses of NAC for APAP therapy, varying from 300 to 1200 mg/kg [12,13,14,15,16].

In our previous studies, we prove that propacetamol, the APAP prodrug, induced similar hepatotoxicity as APAP by similar mechanisms [17,18]. The objectives of the study were: (a) to establish the lethal doses of propacetamol and the optimal therapeutic doses of NAC in two inbred mouse strains; (b) to compare therapeutic effects of different NAC doses in the 1200 mg/kg propacetamol-poisoned mice; (c) to study the molecular mechanisms of NAC overdose in the injury and recovery stages following propacetamol poisoning; (d) to study the toxic effects of NAC overdose in normal mice. Our results can serve as an indispensable reference for determining NAC doses not only in laboratory animals but also in clinical APAP overdose patients and normal persons.

## 2. Materials and Methods

### 2.1. Chemicals and Reagents

Propacetamol was purchased from Standard Chem. & Pharm. (Tainan, Taiwan). NAC, reduced GSH, glutathione peroxidase (GPx), and Tris hydrochloride were obtained from Sigma-Aldrich (St. Louis, MO, USA). Hydrogen peroxide (H_2_O_2_) was purchased from Merck Millipore. Reaction kits for analyses of malondialdehyde (MDA), superoxide dismutase (SOD), and GSH were purchased from Elabscience Biotechnology Inc. (Houston, TX, USA). Tumor necrosis factor (TNF)-α, interleukin (IL)-6, and C-C motif chemokine ligand 2 (CCL2) enzyme-linked immunosorbent assay (ELISA) kits were supplied by BD Bioscience (San Diego, CA, USA). Antibodies to 3-nitrotyrosine (3-NT) (ab7048), goat anti-mouse conjugated horseradish peroxidase, and goat anti-rabbit conjugated horseradish peroxidase antibodies were purchased from Abcam (Cambridge, U.K.). Anti-β-actin monoclonal antibodies (AC-15) were purchased from Novus Biologicals, USA.

### 2.2. Animals and Experimental Design

Five-week-old male BALB/cByJNarl (BALB/c) and C57BL/6JNarl (C57BL/6) mice (weighing 20–22 g each) were purchased from the National Laboratory Animal Center (Taipei, Taiwan). All mice were supplied with sterile water and rodent diet 5001 ad libitum and maintained in a specific pathogen-free environment at the animal center of Chung Shan Medical University under 12 h light/dark cycle, at 22–25 °C with humidity of 50–60%. Animal experimental protocols were in accordance with the guidelines of the institutional animal committees of Chung Shan Medical University (protocol number: 2301). The propacetamol-induced hepatotoxic mouse models were according to our previous reports [17,18]. After adaptation for 1 week, BALB/c and C57BL/6 mice were fasted for 12 h then weighed before experiments. For propacetamol survival studies (total 60 mice), BALB/c mice were randomly divided into two groups (*n* = 10 for each group) according to their body weights to make each group with similar total body weights, intraperitoneally injected with 1200 mg/kg (P1200) and 1400 mg/kg (P1400) propacetamol, and C57BL/6 mice were randomly divided into four groups (*n* = 10 for each group) according to their body weights to make each group with similar total body weights, intraperitoneally injected with P1200, P1400, 1600 mg/kg (P1600), and 1800 mg/kg (P1800) propacetamol, respectively. The survivals of mice were monitored every 2 h for the first 24 h and then every 4 h until 168 h post-propacetamol injection. For the NAC therapy study (total 60 mice), both BALB/c and C57BL/6 mice were randomly divided into six groups (*n* = 5 for each group) according to their body weights to make each group with similar total body weights. They were phosphate-buffered saline (PBS) treatment group (WT), propacetamol treatment group (P1200 equal to 600 mg/kg APAP), and four groups treated with different dosages of NAC (125, 275, 400, and 800 mg/kg) at 1.5 h post-propacetamol injection (P+N125, P+N275, P+N400, and P+N800). For the NAC toxicity study (total of 20 mice), BALB/c mice were divided into four groups (*n* = 5 for each group) according to their body weights to make each group with similar total body weights. They were WT, N275, N400, and N800 groups. Mice were anesthetized with isoflurane (Baxter, CA, USA) before blood collection and tissue analyses at indicated time points. Blood was collected by cardiac puncture for biochemistry and inflammatory factor analyses. The right lobes of the liver were isolated for Hematoxylin and Eosin (H&E) staining and stored at −80 °C for biochemistry and Western blot analyses.

### 2.3. Serum Biochemical Analysis

Blood samples were allowed to sit for 30–40 min before being centrifuged (3000× *g*; 10 min; 4 °C) to collect the serum. Serum was stored at −80 °C and then sent to the National Laboratory Animal Center (Taipei, Taiwan) to analyze serum alanine aminotransferase (ALT) and aspartate aminotransferase (AST) activities [19].

### 2.4. Hematoxylin and Eosin Staining of Liver Sections

The right lobe of the liver was fixed in 10% neutral buffered formalin and embedded in paraffin. Five-micrometer-thick sections were deparaffinized and rehydrated before staining with H&E. Liver histological scoring was performed according to a previously described method [18].

### 2.5. Glutathione and Superoxide Dismutase Levels in the Liver

Liver proteins were prepared by homogenization with PBS, then sonicated and centrifuged to collect the supernatant. The protein concentration was measured by a protein assay reagent (Bio-Rad, Hercules, CA, USA). Reduced GSH levels and SOD activities in the liver were measured using GSH and SOD kits according to the manufacturer’s instructions [20,21].

### 2.6. Malondialdehyde Levels in the Liver

Liver proteins were prepared by homogenization with 1.15% potassium chloride (KCl)/PBS and butylated hydroxytoluene (BHT), then sonicated and centrifuged to collect the supernatant. The MDA level in the liver was measured using an MDA kit according to the manufacturer’s instructions [22].

### 2.7. Glutathione Peroxidase Activity in the Liver

Liver tissues were homogenized with PBS, then sonicated and centrifuged to collect the supernatant. GPx activity was determined according to the method of Lawrence and Burk [23].

### 2.8. Inflammatory Cytokine Levels in Serum and the Liver

Liver proteins were prepared by homogenization with PBS, then sonicated and centrifuged to collect the supernatant. Serum and liver TNF-α, IL-6, and CCL2 levels were measured using ELISA kits according to the manufacturer’s instructions [24].

### 2.9. Western Blotting

Western blotting was performed as previously described [17]. Briefly, liver proteins were prepared by homogenization with radioimmunoprecipitation assay buffer, then sonicated and centrifuged at 10,000× *g* at 4 °C for 20 min. Subsequently, 40 μg of proteins were separated by 12% sodium dodecyl sulfate-polyacrylamide gel electrophoresis and transferred to a polyvinylidene fluoride membrane (Millipore, Billerica, MA, USA) for Western blotting. The membrane was blocked with 5% bovine serum albumin in Tris-buffered saline with Tween 20 (TBST) at room temperature for 1 h, then incubated with primary antibodies against 3-NT (1:3000) and β-actin (1:1000) at 4 °C. This was followed by washing with TBST and incubation with secondary antibodies. β-actin served as the loading control. The immunoreactive bands were visualized with enhanced chemiluminescence substrate (Perkin Elmer, CT, USA), and intensities of bands were quantified using AlphaEaseFC 6.0 software.

### 2.10. Statistical Analyses

Data are expressed as mean ± standard deviation (SD). The mean and SD of targeted analysis levels were calculated for clinical biochemistry, Western blot, and metabolite data using Microsoft Office Excel 2016 software. Statistical analyses were performed using SPSS for Windows, version 17 (SPSS, Inc., Chicago, IL, USA). Data were analyzed with one-way ANOVA followed by Duncan’s multiple range tests. *p* < 0.05 was considered statistically significant. Liver histopathological examination data were analyzed using the Kruskal–Wallis nonparametric test. Kaplan–Meier analysis was applied to the estimation of survival curves and log-rank test to the determination of differences between survival curves.

## 3. Results

### 3.1. Lethal Doses of Propacetamol and Optimal Therapeutic Doses of NAC in Two Inbred Mice

**Figure 1:**
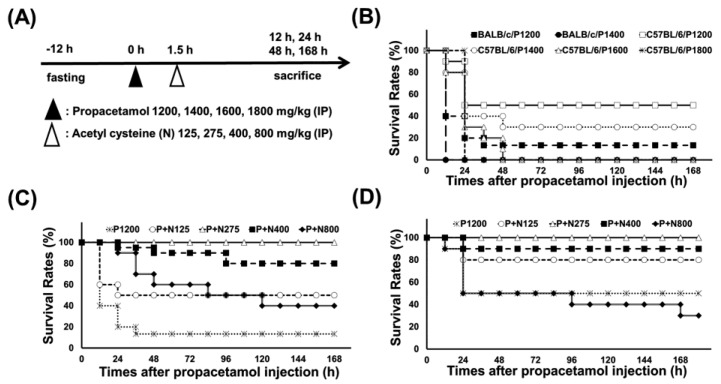
(**A**) Experimental designs and time charts for studying survivals following injections with different propacetamol (P) and N-acetyl cysteine (NAC) (N) doses and the dosage effects of N at indicated time points. (**B**) Survival rates of BALB/cByJNarl (BALB/c) and C57BL/6JNarl (C57BL/6) mice injected with indicated P doses at different time points. The survival rates of BALB/c (**C**) and C57BL/6 (**D**) mice injected with indicated N doses post-1200 mg/kg propacetamol (P1200) injection at different time points.

According to our published researches [17,18], we used propacetamol to establish APAP-poisoning mouse models, followed by treatment with varying doses of NAC at 1.5 h post-propacetamol injection (Figure 1A). Firstly, we treated BALB/c and C57BL/6 mice with different propacetamol doses and observed their survival rates for 7 days. Lethal doses of propacetamol in BALB/c and C57BL/6 mice were 1400 mg/kg (P1400, equal to 700 mg/kg APAP) and 1600 mg/kg (P1600, equal to 800 mg/kg APAP), respectively (Figure 1B). C57BL/6 mice showed higher propacetamol tolerance than BALB/c mice. Most of the propacetamol-poisoned mice died within 48 h, which marked the end of the injury stage and the recovery stage (Figure 1B). P1200 resulted in 10% and 50% survivals of BALB/c and C57BL/6 mice, respectively. This propacetamol dose was selected to analyze the optimal therapeutic dose of NAC in both BALB/c and C57BL/6 mice and the molecular mechanisms of NAC in protecting against propacetamol-induced hepatotoxicity in BALB/c mice. NAC dose of 275 mg/kg (N275) completely rescued propacetamol-poisoned mice with 100% survival of both BALB/c and C57BL/6 mice at 7 days (Figure 1C,D). Surprisingly, higher doses of NAC (N400 and N800) could not rescue all propacetamol-poisoned mice, with some dying after 48 h. The survival rates of the P+N400 and P+N800 groups of BALB/c mice were 80% and 40% at 7 days, respectively (Figure 1C). In the more propacetamol-tolerant mouse strain, C57BL/6, the optimal therapeutic dose of NAC for P1200 was also N275, the same as that of BALB/c mice (Figure 1D). Furthermore, high doses of NAC decreased the survival rates in the P+N400 and P+N800 groups. In the P+N800 group, the survival rate (30%) was even lower than that of the P1200 group (50%) (Figure 1D). Therefore, the optimal NAC therapy dose was 275 mg/kg in both inbred strains of mice for P1200. Higher doses of NAC (N400 and N800) decreased the survival rates of both inbred strains of mice.

**Figure 2:**
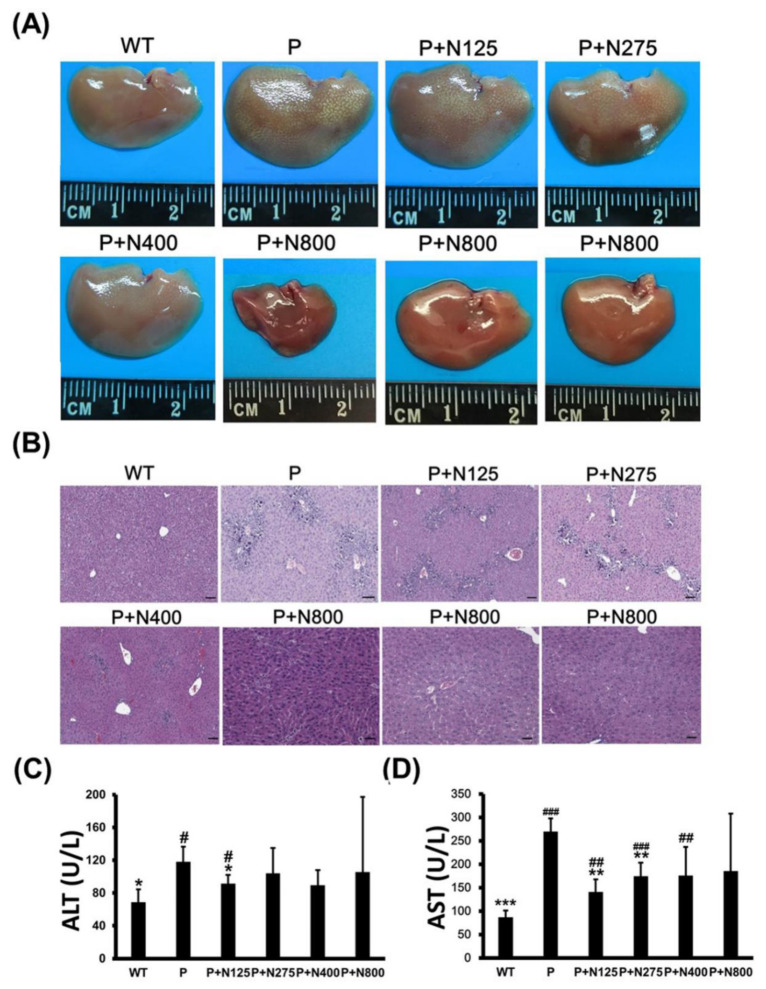
Different NAC doses rescued 1200 mg/kg propacetamol (P)-induced toxicity in BALB/c mice at 7 days. Liver appearances (**A**), histopathological images of liver sections (**B**), and serum alanine aminotransferase (ALT) (**C**) and aspartate aminotransferase (AST) (**D**) activities in surviving mice in indicated groups. Significant differences compared with the P group are indicated by * *(p* < 0.05), ** *(p* < 0.01), and *** *(p* < 0.001). Significant differences compared with the PBS treatment group (WT) group are indicated by # *(p* < 0.05), ## *(p* < 0.01) and ### (*p* < 0.001). Scale bars: 50 μm.

Some mice in the P+N400 and P+N800 groups, but not in the P1200 or P+N125 groups, died after 48 h, which was during the later recovery stage. Thus, we analyzed whether the cause of death in the P+N400 and P+N800 groups was related to liver regeneration between 48 h and 196 h. We compared the appearances and sizes of the livers of surviving mice from different P+N groups at 7 days. Livers in the P+N800 group were smaller than those in the other groups (Figure 2A). H&E staining of liver sections showed that liver tissues had not been completely repaired in any P+N group, except in the P+N800 group. All P+N groups showed better liver regeneration than the P group (Figure 2B). Although mice in the P+N800 group showed almost complete liver repair, some liver cells showed accumulations of lipids and limited gathering of inflammatory cells. Therefore, we suggest that mouse deaths in the P+N400 and P+N800 groups were caused by microvesicular steatosis in the liver and/or inflammation in the late recovery stage rather than by an inability to repair the liver. Serum ALT activities in the P group showed significant increases when compared with the WT group, but ALT activities in all the P+N groups were similar to those in the WT group (Figure 2C). Only the P+N125 group showed a significant decrease in ALT activities compared to the P group. Except for the P+N800 group, serum AST activities in the P and P+N groups showed significant increases when compared with the WT group. However, the P+N125 and P+N275 groups showed significant decreases compared to the P group (Figure 2D). Increases in serum AST activities suggested inflammation and/or damage in all P and P+N groups.

### 3.2. NAC Therapy Effectively Reduces Propacetamol-Induced Acute Liver Injury and Its Side Effects

**Figure 3:**
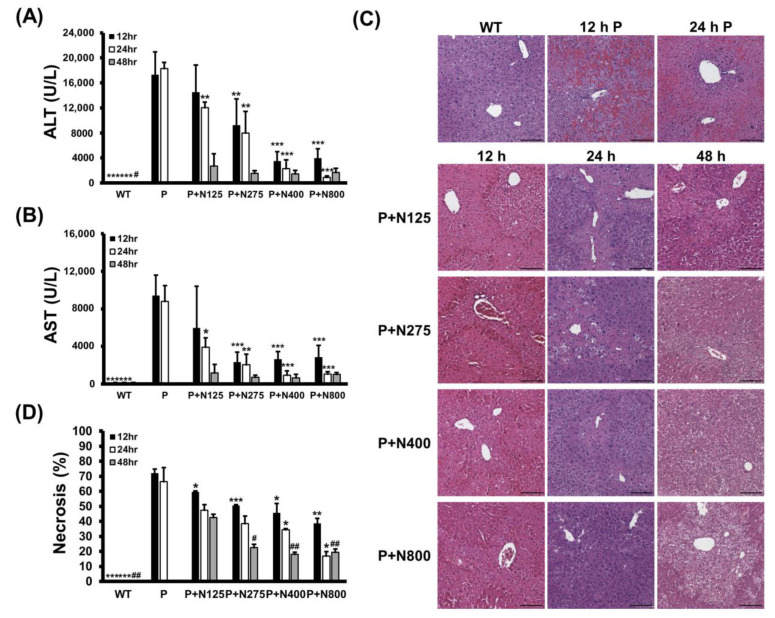
Serum ALT (**A**) and AST (**B**) activities, histopathological images (**C**), and percentages of necrosis (**D**) in liver sections at 12, 24, and 48 h post-P1200 injection and with or without indicated doses of NAC therapies in BALB/c mice. WT: Mice treated with the same volume of phosphate-buffered saline (PBS); P: P1200 injection only; P+N: After P1200 injection, mice were treated with 125 mg/kg (P+N125), 275 mg/kg (P+N275), 400 mg/kg (P+N400), or 800 mg/kg (P+N800) NAC. Significant differences compared with the P group are indicated by * *(p* < 0.05), ** *(p* < 0.01), and *** *(p* < 0.001). Significant differences compared with the P+N125 group are indicated by # *(p* < 0.05) and ## *(p* < 0.01). Scale bars: 100 μm.

We analyzed how different NAC doses affect propacetamol-induced liver damage in the injury and recovery stages in BALB/c mice. Serum ALT and AST activities induced by P1200 injection were significantly lower in the P+N275, P+N400, and P+N800 groups at 12 h and 24 h, but only at 24 h in the P+N125 group when compared with the P group (Figure 3A,B). However, at 48 h, serum ALT and AST activities of all P+N groups were greatly reduced, with no significant differences among these groups. Serum ALT and AST activities in the P+N125 group were higher than those in the other P+N groups at 12 h and 24 h, indicating that this NAC dosage is insufficient to ameliorate propacetamol poisoning in the injury stage, which leads to delayed liver regeneration.

The results of H&E staining showed that propacetamol poisoning induces cell necrosis around the pericentral vein (Figure 3C, 12 h P and 24 h P). This cell necrosis was significantly reduced in both the P+N400 and P+N800 groups at 12 h and 24 h during the injury stage, indicating that an increased dose of NAC decreases hepatocyte injury. Furthermore, the P+N275, P+N400, and P+N800 groups showed significant decreases in percentages of necrosis compared to the P (at 12 h and 24 h) and P+N125 (at 48 h) groups (Figure 3D). There were significant decreases in the percentages of necrosis in the P+N125 group at 12 h, during the injury stage, compared to the P group. However, the P+N125 group showed significant increases of hepatic necrosis compared to the other P+N groups at 48 h, during the recovery stage.

These percentages of necrosis are consistent with the results of serum ALT and AST activities (Figure 3A,B). The higher the dose of NAC, the better the efficacy for reducing propacetamol-induced liver damage at 12 h and 24 h during the injury stage. However, liver damage was more serious, and liver repair was inhibited or delayed in the P+N125 group. As shown in Figure 3, levels of liver damage and recovery in the P+N275 group were similar to those in the P+N400 and P+N800 groups at 48 h. These results supported the survival data (Figure 1C,D) and indicated that 275 mg/kg is the optimal therapeutic dose of NAC for P1200. At 24 h, which marks the end of the injury stage and the beginning of the recovery stage, there were few necrotic cells in the P+N800 group. However, at 48 h, during the recovery stage, there was an obvious accumulation of lipids in hepatocytes of the P+N800 group (Figure 3C, P+N800, 48 h). In combination with our previous results, we suggest that higher doses of NAC (N400 and N800) effectively reduce propacetamol-induced hepatotoxicity and promote liver regeneration. However, they also produce side effects, such as interference with lipid metabolism and additional inflammation, which leads to death during the late recovery stage.

### 3.3. NAC Therapy Effectively Reduces Propacetamol-Induced Oxidative Stress

**Figure 4:**
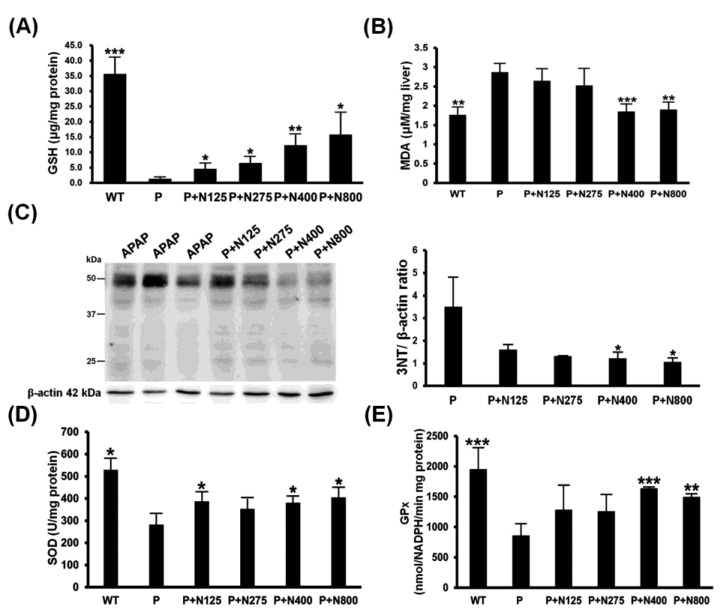
Effects of different doses of NAC therapies on acetaminophen (APAP)-induced oxidative stress in BALB/c mice. The hepatic levels of reduced glutathione (GSH) (**A**), malondialdehyde (MDA) (**B**), nitrotyrosines (**C**), superoxide dismutase (SOD) (**D**), and GPx (**E**) activities were detected in mice at 12 h post-propacetamol injection. Significant differences compared with the P group are indicated by * *(p* < 0.05), ** *(p* < 0.01), and *** *(p* < 0.001).

The main detoxification mechanism of NAC involves providing the materials for the synthesis of GSH to neutralize the NAPQI produced during APAP metabolism, thereby reducing the APAP-induced oxidative stress. Our results showed that hepatic reduced GSH levels were almost exhausted in the P group. NAC therapy showed dosage-dependent increases in hepatic GSH levels (Figure 4A). Furthermore, propacetamol-induced lipid oxidation (MDA) and protein nitration (3-NT) significantly decreased in the P+N400 and P+N800 groups compared to the P group (Figure 4B,C).

The hepatic activities of two major antioxidative enzymes, SOD and GPx, were significantly decreased in the P group compared to the WT group and then significantly increased in the P+N400 and P+N800 groups compared to the P group (Figure 4D,E). Therefore, higher doses of NAC (P+N400 and P+N800) significantly reduced propacetamol-induced oxidative stress through increased hepatic levels of reduced GSH and recovered antioxidative enzyme activities.

### 3.4. NAC Therapy Effectively Reduces Propacetamol-Induced Inflammatory Cytokines

**Figure 5:**
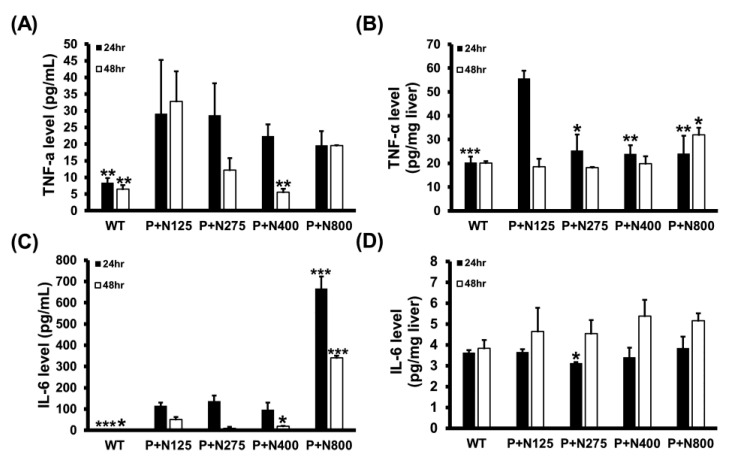
Effects of different doses of NAC therapies on serum (**A**,**C**) and hepatic (**B**,**D**) levels of tumor necrosis factor (TNF)-α (A,B) and IL-6 (**C**,**D**) at 24 h and 48 h post-propacetamol injection in BALB/c mice. Significant differences compared with the P+N125 group are indicated by * (*p* < 0.05), ** *(p* < 0.01), and *** *(p* < 0.001).

We analyzed the serum and hepatic levels of two major inflammatory cytokines, TNF-α and IL-6, in mice of the WT and P+N groups. Compared to the P+N125 group, higher doses of NAC therapies reduced serum TNF-α levels at 48 h and significantly decreased hepatic TNF-α levels at 24 h (Figure 5A,B). However, a significant increase in hepatic TNF-α levels was observed in the P+N800 group compared to the P+N125 group at 48 h. Moreover, compared to the P+N125 group, we observed significant increases in serum IL-6 levels in the P+N800 group at both 24 h and 48 h and no significant difference in hepatic IL-6 levels in the P+N400 and P+N800 groups at 48 h (Figure 5C,D). Serum and hepatic levels of inflammatory cytokines indicated systemic and hepatic inflammations in all NAC therapy groups at 24 h during the injury stage. Both systemic and hepatic inflammations were observed only in the P+N800 group at 48 h during the recovery stage. Since TNF-α and IL-6 also stimulate signaling pathways contributing to liver regeneration post-APAP poisoning [25], we analyzed the cyclin D1 levels in mouse livers of the WT and P+N groups at 48 h post-propacetamol injection (Figure S1). These results show significant increases in hepatic cyclin D1 levels in all P+N groups than the WT group, but no significant difference was found between all P+N groups (Figure S1). Thus, elevated levels of NAC do not show a significant effect on hepatic cyclin D1 levels in the recovery stage.

### 3.5. High-Dose NAC Treatment Reduces Hepatic GSH Levels and Survival Rates in Mice

**Figure 6:**
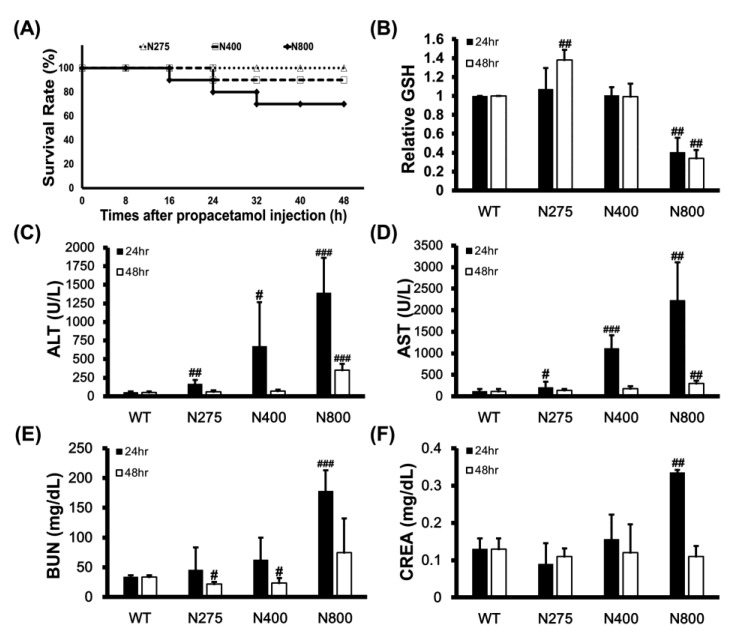
Effects of different doses of NAC on survival rates of BALB/c mice within 48 h (**A**), relative reduced GSH levels in liver (**B**), serum ALT (**C**) and AST (**D**) activities, and blood urea nitrogen (BUN) (**E**) and creatinine (CREA) (**F**) levels at 24 h and 48 h post-NAC injection. The GSH levels in the WT group were defined as 100%. Significant differences compared with the WT group are indicated by # *(p* < 0.05), ## *(p* < 0.01), and ### *(p* < 0.001).

We examined the mortality effects of different doses of NAC on normal mice to determine whether high-dose NAC (N800) causes dramatic increases in serum IL-6 levels, induces systemic and hepatic inflammations, and decreases the survival of mice. High doses of NAC, specifically N400 and N800, reduced survival rates of mice to 90% and 70% at 48 h, respectively (Figure 6A). This result supports that high-dose NAC (P+N400 and P+N800) decreases mouse survival rates (Figure 1C,D). Since NAC provides the raw materials for GSH synthesis, we analyzed the hepatic levels of reduced GSH in different NAC dosage groups. Surprisingly, only the N275 group showed significantly increased hepatic GSH levels at 48 h. When compared with the hepatic GSH levels in the WT group, those in the N800 group significantly decreased at both 24 h and 48 h post-NAC injection (Figure 6B). Furthermore, we found that the hepatic GSH levels in all the P+N groups were lower than the WT group, and only in the P+N125 and P+N800 groups were significantly lower than those in the P+N275 group at 48 h during the liver recovery stage (Figure S2).

Since intraperitoneally injected NAC is rapidly absorbed by and distributed in the liver, kidney, and duodenum, we analyzed the impacts of different doses of NAC on mouse liver and kidney functions. Serum ALT and AST activities that are liver damage markers significantly increased with increasing doses of NAC at 24 h, then recovered to levels similar to those of the WT group at 48 h in both the N275 and N400 groups, but not in the N800 group (Figure 6C,D). Serum blood urea nitrogen (BUN) and creatinine (CREA) levels that are kidney damage markers also significantly increased in only the N800 group at 24 h but decreased to normal levels compared to the WT group (Figure 6E,F). These results indicated that high NAC doses (N400 and N800) in normal mice damage liver and kidney (N800 only), especially at 24 h post-NAC injection. In combination with the results shown in Figure 6B and Figure S2, we conclude that high NAC doses decrease hepatic GSH levels, induce liver and kidney damages, and may result in death.

### 3.6. High-Dose NAC Treatment Increases Serum Levels of Inflammatory Cytokines

**Figure 7:**
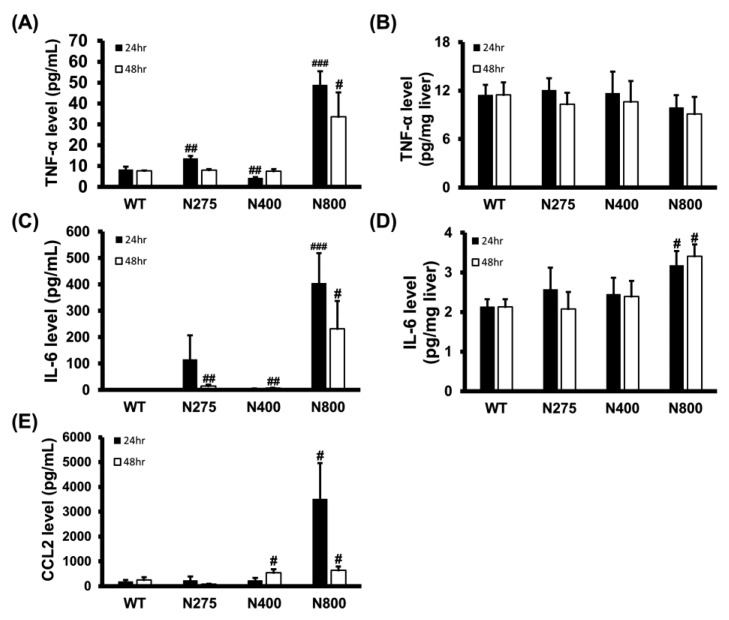
Effects of different doses of NAC administered to BALB/c mice on serum (**A**,**C**,**E**) and hepatic (**B**,**D**) levels of TNF-α (**A**,**B**), IL-6 (**C**,**D**), and CCL2 (**E**) at 24 h and 48 h post-NAC injection. Significant differences compared with the WT group are indicated by # *(p* < 0.05), ## *(p* < 0.01), and ### *(p* < 0.001).

As shown in Figure 5, the serum and hepatic levels of inflammatory cytokines were influenced by NAC therapy. We analyzed the serum and hepatic levels of inflammatory cytokines in normal mice treated with different doses of NAC. Serum TNF-α, IL-6, and CCL2 levels dramatically increased at both 24 h and 48 h post-NAC injection in the N800 group compared to the WT group (Figure 7A,C,E). We also observed significant increasein serum TNF-α and IL-6 levels in the N275 group and IL-6 and CCL2 levels in the N400 group at 24 h and/or 48 h. However, except for the N800 group in IL-6 levels, there were no significant differences in hepatic TNF-α and IL-6 levels among the tested groups (Figure 7B,D). As shown in Figure 5 and Figure 7, high doses of NAC, especially N800, dramatically increased serum levels of inflammatory cytokines, resulting in an immune storm that may lead to deaths in both the P+N800 and N800 groups.

### 3.7. High-Dose NAC Treatment Induces Microvesicular Steatosis in Liver

**Figure 8:**
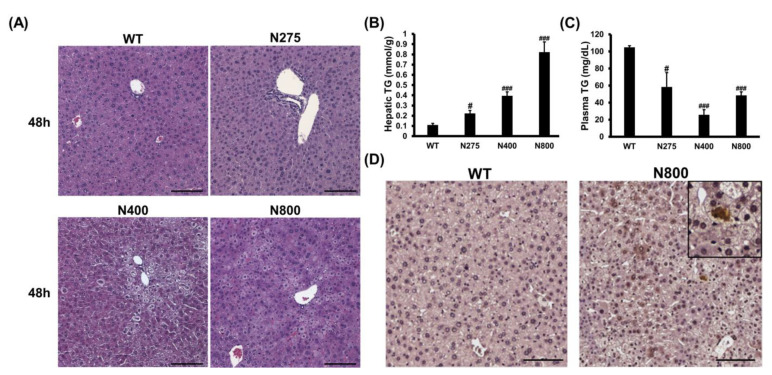
Effects of different doses of NAC on histopathological images (**A**), hepatic and serum triglyceride (TG) levels (**B,C**), and IL-6 immunohistochemistry images of liver sections (**D**) at 48 h post-NAC injection in BALB/c mice. Significant differences compared with the WT group are indicated by # *(p* < 0.05) and ### *(p* < 0.001). Scale bars: 100 μm.

Since high-dose NAC (N800) decreases hepatic reduced GSH levels, increases hepatic IL-6, and damages mouse liver (Figure 6 and Figure 7), we attempted to decipher how NAC affects the liver in normal mice. In both the N400 and N800 groups, the liver appeared dehydrated at 48 h (Figure S3A). Lipid accumulations in hepatocytes, known as the fatty liver phenomenon, were observed in both the N400 and N800 groups at 48 h (Figure 8A). From our data, 1 of 5 mice in the N400 group and 4 of 5 mice in the N800 group showed fatty liver phenomenon. Hepatic triglyceride (TG) levels were significantly increased in all the N275, N400, and N800 groups at 48 h post-NAC injection compared to the WT group (Figure 8B). However, serum TG levels in all the N275, N400, and N800 groups showed a significant decrease at 48 h compared to the WT group (Figure 8C). Furthermore, serum total cholesterol (T-CHO) levels significantly increased in the N800 group at 48 h compared to the WT group (Figure S3B). These results supported the findings of fatty liver in the N800 (Figure 8A) and P+N800 groups (Figure 2B and Figure 3C).

As hepatic IL-6 levels slightly increased and serum IL-6 levels significantly increased in the N800 group (Figure 7C,D), we detected the hepatic IL-6 expression in the WT and N800 groups at 48 h by immunohistochemistry. The IL-6 expressions were detected in liver sections of the N800 group with steatosis, mainly localized in hepatocytes (Figure 8D). In addition, serum CCL2 levels dramatically increased in the N800 group at both 24 h and 48 h (Figure 7E). From the above results, we suggest that high doses of NAC, especially the N800 and P+N800, induce systemic inflammation and interfere with liver metabolism, resulting in fatty liver and even death in mice. Furthermore, high doses of NAC may interfere with hepatic GSH levels and damage both the liver and kidneys in normal mice.

## 4. Discussion

In addition to being used to treat acute liver injury in APAP overdose, NAC is also used to treat the deterioration of chronic obstructive pulmonary disease [26]. Recent studies have also tried to use NAC to treat neurological diseases and metabolic-related diseases [27,28]. Although NAC is considered to be a multifunctional therapeutic drug, it mainly functions as an antioxidant and replenishes the GSH-less cells that are depleted by oxidative stress, but it may not be effective for GSH-rich cells and may even become a pro-oxidant [29]. However, the toxic mechanism of NAC overdose for sick or healthy people is not clear. Clinically, NAC overdose treatment for APAP overdose patients leads to death [9]. Therefore, the main objective of this study is to explore the toxic effects of NAC overdose on propacetamol-induced liver-damaged and normal mice. The results show that overdose of NAC has a significant risk of inducing fatty liver and systemic inflammation, and severe cases can be fatal.

NAC standard treatment is not suitable for all patients. Comparative clinical trials have been insufficient to determine the optimal NAC therapeutic dose [30]. Therefore, whether increasing the therapeutic dose of NAC is helpful for APAP fatal liver injury must be deciphered in the mouse model. However, in the literature, doses of APAP and NAC used in mouse models have varied among laboratories [31,32]. Therefore, we established the APAP lethal doses in two commonly used inbred mouse strains, C57BL/6 and BALB/c. Inbred mouse strains are used to test efficacies or toxicities of drugs because most of their genetic lineages are known, and their numbers of genetic polymorphisms are equal to or greater than those of the human population [33].

The results of survival rates show that BALB/c mice are more sensitive than C57BL/6 mice to propacetamol-induced liver failure. This is consistent with the results of Harrill et al. [33], who reported liver necrosis ratios of between 30% and 60% in BALB/c mice and between 20% and 30% in C57BL/6 mice at 24 h post-APAP injection. Furthermore, a high degree of intra-strain variability in APAP-induced toxicity has been reported in C57BL/6 mice [34].

Next, is the optimal therapeutic dose of NAC for the same APAP dose also different in different susceptibility mice? Although BALB/c and C57BL/6 mice showed different sensitivities to propacetamol, N275 completely rescued the toxicity of P1200 (equal to 600 mg/kg APAP) in both mouse strains for 7 days. Surprisingly, mice of both strains in the high-dose NAC therapy groups (P+N400 and P+N800) died between 48 h and 120 h post-propacetamol injection. It shows that elevating NAC dose cannot increase the therapeutic effect, but on the contrary, produces fatal side effects. This result is consistent with the death caused by clinical NAC overdose treatment in APAP poisoning patients [35].

Studies have shown that the first mechanism for NAC therapy to treat APAP overdose hepatotoxicity is to provide substances that synthesize GSH to neutralize APAP-derived NAPQI. The second mechanism is that NAC can also increase the GSH levels of mitochondria to protect against oxidative stress and peroxynitrite formation resulting from mitochondrial dysfunction [6]. According to the survival rate in BALB/c mice, P1200 caused great death within 12 h, resulting in a survival rate of 40% at 12 h. Therefore, we analyzed the effects of different NAC doses on reducing propacetamol-induced oxidative stress at 12 h post-propacetamol injection. As the dose of NAC increased, propacetamol-induced liver damages were decreased, as evidenced by decreasing serum ALT/AST activities and histopathological scores. NAC therapy shows dosage-dependent increases of hepatic reduced GSH levels, which was exhausted by APAP overdose. NAC treatments significantly decreased propacetamol-induced oxidative stress and peroxynitrite formation (MDA and 3-NT formations). Furthermore, NAC therapy also decreased the propacetamol-induced oxidative stress through reversing activities of the propacetamol-decreased antioxidant enzymes, SOD, and GPx. These results show that increasing the NAC dose (from N125 to N400) improves the hepatic protective effects, similar to the report by Saito et al. (from NAC106 to NAC318) [6].

It is known that APAP induces early cell death, then induced inflammatory response to recruit neutrophils for removing necrotic cells but not cause additional damage [36]. The inflammation response occurs slightly later than the injury stage by a substantial increase in serum TNF-α level at 24 h [37]. APAP also induces other inflammatory cytokines, including IL-1β, IL-6, macrophage inflammatory protein 2 (MIP-2), and CCL2. NAC treatment also decreases these inflammatory factors [13]. Our results also showed the abilities of NAC treatments in reducing propacetamol-induced hepatic and serum TNF-α levels at both 24 h and 48 h, consistent with the report by James et al. [13].

However, the function of IL-6 is recognized to be related to the regulation of acute inflammation response [38]. The propacetamol-induced serum IL-6 levels were decreased from 24 h to 48 h following NAC therapy, similar to that of serum TNF-α levels, indicating that the propacetamol-induced liver injury decreased from 24 h to 48 h. However, IL-6 is a pleiotropic cytokine, and a previous study has shown that IL-6 is also related to liver repair and regeneration [39]. In our data, the hepatic IL-6 levels in all the NAC therapy groups increased from 24 h to 48 h, parallel to the liver regeneration stage, indicating hepatic cell proliferation. However, the serum IL-6 levels in the P+N800 group were much higher than the other P+N therapy groups at both 24 h and 48 h. In addition, N800 treatment also induced serum levels of TNF-α and dramatically high levels of serum IL-6 and CCL2 in normal mice at both 24 h and 48 h. The N800 treatment induced high levels of serum CCL2 and IL-6, accompanied by the appearance of steatosis in both propacetamol-poisoned and normal mice. Furthermore, the N800-induced steatosis was still present at 7 days post-propacetamol injection. A small number of hepatocytes showed lipid accumulation, and livers in some mice of the P+N800 therapy group were generally smaller than in those of the other P+N therapy groups. We speculated that although NAC neutralizes the toxins produced by APAP, at the same time, NAC overdose (N800) induces systemic inflammation and steatosis in APAP disease mice.

Microvesicular steatosis was more severe in the P+N800 therapy group than in the N800 treatment group. This may be due to APAP-induced mitochondrial dysfunction, which led to both centrilobular necrosis and microvesicular steatosis. Since microvesicular steatosis is mainly caused by severe impairment of mitochondrial fatty acid β-oxidation, it can be life-threatening [40,41,42]. In addition, clinically, microvesicular steatosis is associated with an increase in serum aminotransferase levels and mitochondrial dysfunction [43]. In our study, serum ALT/AST activities were significantly induced in the N800 treatment group at both 24 h and 48 h and associated with microvesicular steatosis. We speculate that NAC-induced microvesicular steatosis in the P+N800 and N800 groups was associated with mitochondrial dysfunction and may be life-threatening when extensive or lasting.

There is little evidence to indicate that NAC induced system inflammation. Injection of NAC irritates the peritoneal lining and decreases mucosal GSH [44]. Furthermore, in the chemical-induced colitis mouse model, the inflammation of mesenteric fat was more severe than that of liver [45]. Therefore, we speculated that a high NAC dose induces systemic inflammation partly due to direct irritation of the peritoneal lining, with intestinal inflammation resulting in mesenteric fat inflammation, steatosis, and hepatic inflammation. The exact mechanism of high-dose NAC-induced steatosis and inflammation in normal mice needs to be deciphered at more than 48 h post-NAC injection in the future.

There is no evidence regarding the side effects of high-dose NAC in healthy persons. However, in the animal model, repeated oral administration of NAC in rats significantly elevated serum ALT activities [46]. The above result is consistent with our results. N800 treatment induced significant increases of both serum ALT/AST values at 24 h, although recovered at 48 h, still higher than those of the WT group. In combination with our results and the previous report, no matter what it is, one-injection or long-term administration of NAC, NAC overdose induces liver damage proved by increases of serum ALT/AST activities. Furthermore, NAC overdose also affects kidney function, proved by increases in serum BUN/CREA levels in this study. Serum BUN and creatinine (CREA) levels significantly increased only in the N800 group at 24 h but decreased to normal levels at 48 h compared to those of the WT group. At 48 h, mice in the N800 group show signs of weakness and reduced mobility. Mice in the N800 group are difficult to obtain blood samples at 48 h post-NAC injection. The significant increases then decrease in serum BUN and creatinine levels in mice of the N800 group may reflect those mice that are dying or with contracted vascular space, as reflected by reduced survival and difficulty in obtaining blood samples. Excessive use of NAC in a short period of time also leads to hemolysis and acute renal failure in patients [9]. Therefore, we suggest that acute NAC overdose induces both liver and kidney damage in rodents and human patients.

The main function of NAC is to provide raw materials for cells to synthesize GSH. According to Zwingmann et al. [47], GSH synthesis in the liver reached a peak at 300 mg/kg NAC, which is consistent with our results that hepatic GSH levels in the N275 and N400 treatment groups are higher than or equal to that in the WT group. Therefore, NAC shows a limited capacity to induce de novo synthesis of GSH in mouse liver, and 275–400 mg/kg NAC is the peak range. However, NAC overdose decreases hepatic GSH levels at both 24 h and 48 h post-NAC injection in this study and in repeated oral administration of NAC in rats [46]. Since NAC is a reactant of mitochondrial energy metabolism, high doses of NAC (600 to 1200 mg/kg) have been reported to inhibit pyruvate carboxylase (PC) and promote pyruvate dehydrogenase (PDH) in the tricarboxylic acid cycle [47]. In addition, PC inhibition leads to decreases in glutamate and GSH levels and results in a more oxidized redox state in PC knockout mice [48]. Therefore, we speculated that one of the ways to reduce GSH level by high-dose NAC (N800) might be to inhibit PC activities. Decreased GSH levels in the fatty liver were likely due to ROS resulting from lipid peroxidation [49]. In our study, high-dose NAC (N800)-induced hepatic steatosis also consumed GSH in both normal and propacetamol-poisoned mice, resulting in low hepatic GSH levels.

In our study, medium doses of NAC (N275 and N400), especially N400, significantly reduced serum TG levels in normal mice. NAC has also been reported to reduce blood TG in WT mice [50]. However, in our study, NAC overdose (N800) induced liver steatosis and dramatically increased hepatic TG levels at 48 h post-NAC injection. On the other hand, NAC has been reported to block hepatic lipid accumulation in preclinical models of NAFLD [51]. In rodent disease models, NAC neutralizes oxidative stress by providing GSH, thereby reducing the development of diseases. In normal mice of this study, high-dose NAC reduced hepatic GSH levels, induced liver damage, and then increased serum ALT/AST activities. It has also been reported that NAC is an antioxidant but shows pro-oxidant activity and causes hypotension [29]. Thus, NAC is not recommended for patients without oxidative stress. In addition, the dosage of NAC must be carefully evaluated, especially in the presence of diseases, such as APAP-induced liver injury and metabolic abnormality, to prevent the additive effect of NAC toxicity. Furthermore, it is important to decipher the mechanisms from which high-dose NAC induces microvascular steatosis.

Treatment of APAP toxicity at a dose greater than 3000 mg/person/day NAC results in allergic reactions, including rashes, bronchospasms, and hypotension. According to the 12.3-fold metabolic conversion factor, this is equivalent to about 615 mg/kg/day NAC in mice [52]. This dose was considered toxic in this study. In our mouse model, bodyweights of mice were significantly reduced in all the P+N therapy and NAC treatment groups at both 24 h and 48 h compared to the WT group (Figure S4). There were significant bodyweight losses after 48 h of NAC treatments (Figure S4 panel B). However, only high doses of NAC (400 and 800 mg/kg) induced steatosis. Mice in the P+N400 and P+N800 groups lost more bodyweight at 48 h than those in the other groups (Figure S4 panel A), but these mice showed better liver repair than those of the P+N125 and P+N275 groups. These results indicate that therapies with high doses of NAC show better therapeutic effects for propacetamol-induced hepatotoxicity. However, high doses of NAC cause systemic damage, affect overall physiological functions, and cause physical weakness and even death in mice. Although some side effects induced by NAC such as persistent vomiting or allergic reactions in humans are not easy to detect in mice, other NAC-induced side effects including unusual weakness, abnormal appearance at the injection site, abnormal liver function, decrease in food uptake (gastrointestinal disturbances), and difficulty in drawing blood (heart and blood vessel abnormalities) are found in both humans and mice. Many physiological functions or responses of mice to NAC overdose are similar to those of humans. Therefore, this study showing the toxicity of NAC overdose in mice will provide a reference for the NAC dose used in humans, although the doses used in mice and in humans cannot be directly converted.

## 5. Conclusions

In conclusion, this study proves that BALB/c inbred mice are more sensitive than C57BL/6 inbred mice to propacetamol-induced hepatotoxicity. The optimal therapy dose of NAC is 275 to 400 mg/kg for 1200 mg/kg propacetamol injection (equal to 600 mg/kg APAP) in both inbred mouse strains with different propacetamol sensitivities. NAC doses exceeding 400 mg/kg reduce liver GSH levels, induce steatosis and systemic inflammation, and may lead to death in normal and propacetamol-poisoned mice.

## Data Availability

The data presented in this study are available on request from the corresponding author.

## Supplementary Materials

The following are available online at https://www.mdpi.com/2076-3921/10/3/442/s1. Figure S1: High-dose NAC treatment reduces hepatic GSH levels. Figure S2: High-dose NAC treatment induces serum T-CHO in the liver. Figure S3: High-dose NAC treatment induced bodyweight losses.
