# Supplementary material for: N-Acetyl Cysteine Overdose Inducing Hepatic Steatosis and Systemic Inflammation in Both Propacetamol-Induced Hepatotoxic and Normal Mice
Source: Antioxidants (Basel). 2021 Mar 12;10(3):442. doi: 10.3390/antiox10030442 (PMC8000488; doi:10.3390/antiox10030442)
Supplement: Supplementary file 1 [file antioxidants-10-00442-s001.pdf]

Supplementary Figures:

Figure S1: Elevated levels of NAC do not show significant effect on hepatic cyclin D1 levels in recovery stage.

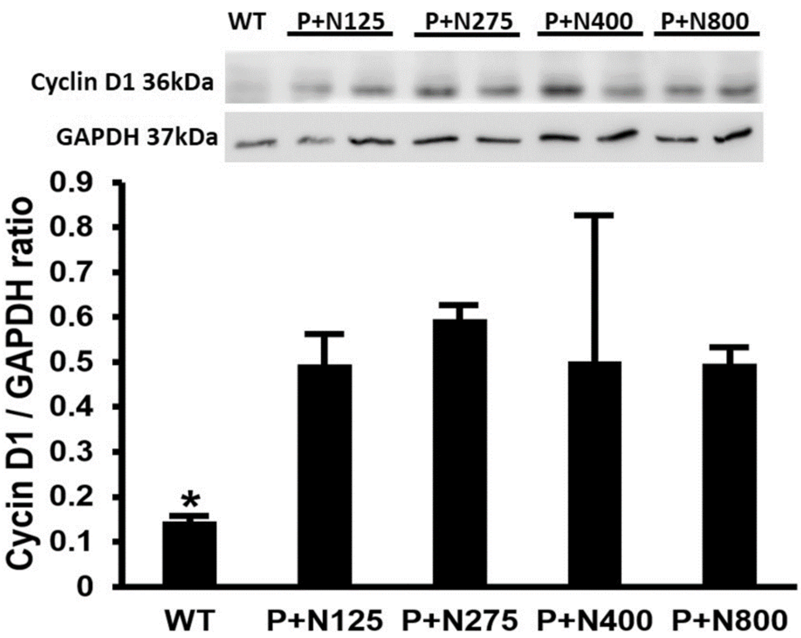

Figure S1. Effect of different doses of NAC therapy on cyclin D1 levels in BALB/c mice at 48 h post-propacetamol injection. Significant differences compared with the P+N125 group are indicated by * (P < .05).

Figure S2: High-dose NAC treatment reduces hepatic GSH levels

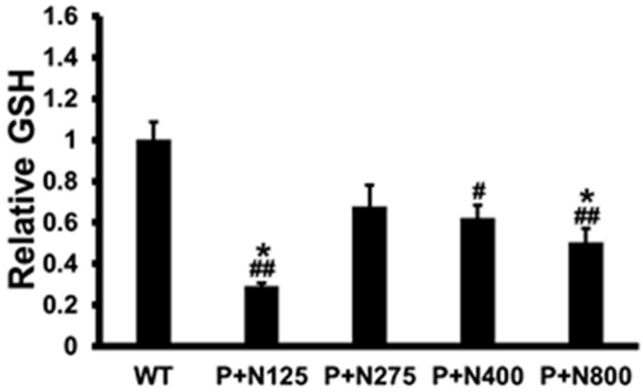

Figure S2. Effects of different doses of NAC on hepatic reduced GSH levels in BALB/c mice at 48 h post-propacetamol injection. The GSH levels in the WT group were defined as 100%. Significant differences compared with the WT group are indicated by # (P < .05) and ## (P < .01). Significant differences compared with the P+N275 group are indicated by * (P < .05).

Figure S3: High-dose NAC treatment induces serum T-CHO levels in liver

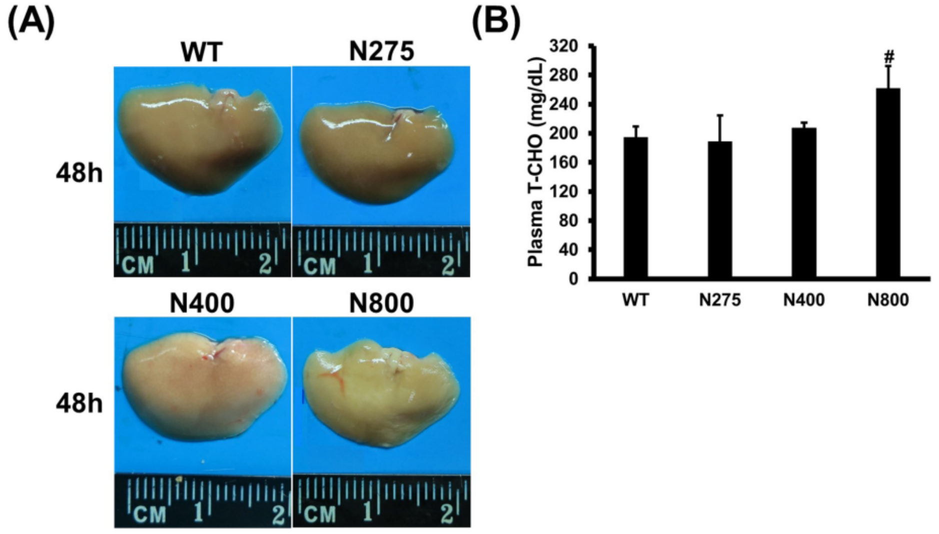

Figure S3. Effects of different doses of NAC on liver appearances (A) and serum T-CHO levels (B) in BALB/c mice at 48 h post NAC-injection. Significant difference compared with the WT group is indicated by # (P < .05).

Figure S4: High-dose NAC treatment induced body weight losses

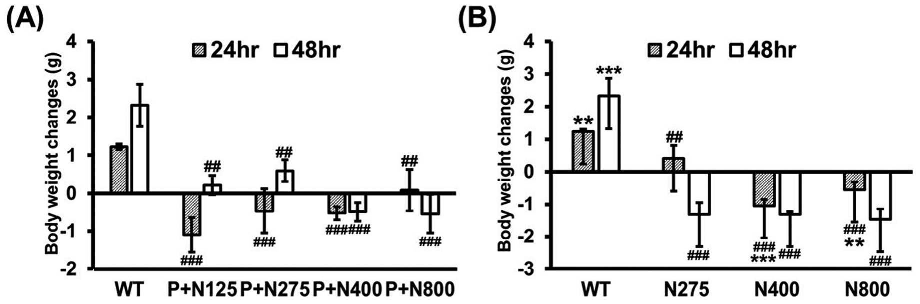

Figure S4. Body weight changes in BALB/c mice of different doses of the NAC therapy (A) and the NAC treatment (B) groups at indicated time points. Significant differences compared with the WT group are indicated by # (P < .05), ## (P < .01) and ### (P < .001). Significant differences compared with the N275 group are indicated by ** (P < .01) and *** (P < .001).
